# Susceptibility of *Staphylococcus aureus* Strains Isolated from Blood Cultures Between 2024 and 2025: A Single-Center Study

**DOI:** 10.3390/antibiotics15070695

**Published:** 2026-07-16

**Authors:** Victoria Birlutiu, Olteanu Ciprian Ion, Rares-Mircea Birlutiu

**Affiliations:** 1 Faculty of Medicine, Lucian Blaga University of Sibiu, Str. Lucian Blaga, Nr. 2A, 550169 Sibiu, Romania; victoria.birlutiu@ulbsibiu.ro; 2 County Clinical Emergency Hospital, Bvd Corneliu Coposu, Nr. 2–4, 550245 Sibiu, Romania; 3 Faculty of Medicine, The “Carol Davila” University of Medicine and Pharmacy, 050474 Bucharest, Romania; 4 “Foisor” Clinical Hospital of Orthopaedics, Traumatology and Osteoarticular TB, 021382 Bucharest, Romania

**Keywords:** blood culture, *Staphylococcus aureus*, AST, susceptibility

## Abstract

Background: *Staphylococcus aureus* bloodstream infection remains a major clinical challenge because of its association with invasive disease, antimicrobial resistance, biofilm formation, and substantial mortality. Local susceptibility surveillance is essential for guiding empirical therapy and antimicrobial stewardship. This study evaluated the epidemiology, resistance patterns, turnaround time, and clinical correlates of *S. aureus* bacteremia in a Romanian tertiary-care hospital. Methods: We conducted a retrospective, single-center observational study including all consecutive adults with positive blood cultures for *S. aureus* admitted to Sibiu County Clinical Emergency Hospital between 1 January 2024 and 31 December 2025. Demographic, clinical, microbiological, antimicrobial susceptibility, and outcome data were extracted from electronic medical records. MRSA was defined phenotypically, while MDR and XDR phenotypes were classified according to standard resistance definitions. Between-year comparisons and regression analyses were performed to explore factors associated with mortality, MRSA, and MDR status. Results: During the study period, 11,578 blood culture sets were processed, of which 2472 (21.3%) were positive; *S. aureus* accounted for 314 isolates (12.7% of positive cultures). After one isolate per patient was retained, the final analysis included 142 patients with *S. aureus* bacteremia. Median age was 70 years, 55.6% were male, and 67.6% lived in urban areas. Overall mortality was 33.1% (47/142). MRSA was identified in 39.4% of cases and MDR in 49.3%, while no XDR isolates were detected. The most frequent resistances were observed for penicillin (75.9%), erythromycin (66.2%), clindamycin (46.5%), tetracycline (45.1%), and oxacillin (38.0%). Full susceptibility was preserved for vancomycin, linezolid, ceftaroline, daptomycin, and mupirocin. Turnaround time significantly improved in 2025 compared with 2024 (median 71.4 h vs. 66.3 h; *p* = 0.004). In adjusted analysis, acute or chronic kidney disease was independently associated with mortality, whereas MRSA and MDR status were not independent predictors of death. Conclusions: In this single-center cohort, *S. aureus* bacteremia was associated with high mortality and a substantial burden of MRSA and MDR phenotypes, although preserved activity of key anti-staphylococcal agents was observed. These findings support the need for continuous local resistance surveillance, rapid microbiological diagnosis, and stewardship-guided treatment strategies.

## 1. Introduction

*Staphylococcus aureus* (*S. aureus*) is a major human pathogen responsible for a broad spectrum of clinical manifestations, ranging from localized infections to severe systemic disease, including sepsis, infective endocarditis, and bronchopneumonia. It is implicated in both community-acquired and healthcare-associated infections and continues to pose significant therapeutic challenges, largely due to the ongoing increase in antimicrobial resistance.

*S. aureus* ranks among the leading causes of bloodstream infections, second only to Escherichia coli, and has been reported to account annually for approximately 10–30 cases per 100,000 inhabitants [1,2], while mortality among hospitalized patients remains substantial, ranging between 15% and 40% [3,4]. In response to the increasing global burden of antimicrobial resistance, the World Health Organization has included *S. aureus* among priority bacterial pathogens [5], particularly because of the rising prevalence of methicillin-resistant *S. aureus* (MRSA), which has been reported to reach approximately 35% in certain healthcare settings [6].

According to ECDC reports, Europe recorded a 20.4% decrease in MRSA bloodstream infections in 2024 compared with 2019, reaching an overall rate of 4.48 cases per 100,000 population [7]. However, the same source reported a 32.6% increase in *S. aureus*-positive blood culture cases in the EU/EEA, rising from 74,180 in 2020 to 98,335. In Romania, 1028 cases of *S. aureus* infections were reported in 2024 among patients hospitalized in intensive care units, accounting for 19% of all isolates. This represented an increase compared with the 2020–2023 period. MRSA accounted for 13.15% of *S. aureus*-positive blood cultures, increasing from 9.03% in 2020 to 11.99% in 2023.

*S. aureus* combines antimicrobial resistance with several immune-evasion strategies. Methicillin resistance reduces the activity of most beta-lactams, although susceptibility is not uniform: the fifth-generation anti-MRSA cephalosporins ceftaroline and ceftobiprole usually retain activity, and phenotypes such as MODSA and BORSA, together with heterogeneous mecA expression, blur the resistant/susceptible boundary. Reduced susceptibility to glycopeptides (vancomycin, teicoplanin) and to oxazolidinones such as linezolid is also described, though it remains uncommon. Beyond resistance, the organism forms biofilm on endocardial surfaces and indwelling devices, which supports persistence, complicates treatment, and predisposes to recurrent or complicated bloodstream infection [8,9]; this ability also facilitates persistence on hemodialysis catheters, ventriculoperitoneal shunts, and various other implantable medical devices, as well as intracellular survival within endothelial cells, epithelial cells, and osteoblasts [10].

The resistance profile differs between community-associated MRSA (CA-MRSA) strains, which are typically resistant to beta-lactams and may harbor specific virulence factors such as Panton–Valentine leucocidin [11], and healthcare-associated MRSA (HA-MRSA) strains [12], which are more frequently characterized by multiple resistance mechanisms and an increased risk of invasive infections. In practice, however, the epidemiological and genetic distinction between the two has blurred over the past decade, with community strains increasingly recovered in hospitals and healthcare-associated strains in the community.

Selective pressure from fifth-generation cephalosporins may also drive modified *S. aureus* (MODSA) phenotypes with reduced penicillin-binding-protein affinity [13].

*S. aureus* can also acquire fusidic acid resistance, chiefly through alterations in elongation factor G (fusA) or acquisition of FusB-family protection proteins [14].

Other risk factors for *Staphylococcus aureus* bacteremia, beyond antimicrobial resistance itself, include extremes of age, diabetes mellitus, HIV infection, chronic kidney disease, intravenous drug use, and underlying cardiovascular disease [15]. Early recognition of sepsis, particularly infective endocarditis, together with prompt identification of the primary infectious focus, which may sometimes be difficult to detect, such as in osteomyelitis or spondylodiscitis, is essential, as delays in diagnosis may adversely affect prognosis. Inadequate first-line antimicrobial therapy may further contribute to unfavorable outcomes. Therefore, knowledge of local epidemiology and territorial antimicrobial susceptibility patterns is critical for guiding appropriate empirical and targeted therapy, which is consistently associated with lower mortality in *S. aureus* bacteremia [16].

Blood culture remains the gold standard for confirming systemic infections caused by *S. aureus*. Unlike coagulase-negative staphylococci, for which positive blood cultures may frequently reflect contamination, the isolation of *S. aureus* from blood should be regarded as clinically significant, with contamination being exceptional. *S. aureus* bacteriuria may also represent an important microbiological clue to underlying *S. aureus* bacteremia.

Regardless of the primary focus of bacteremia, the identification of a positive blood culture for *S. aureus* should prompt careful assessment and source control. This includes removal of infected or potentially colonized devices whenever feasible, as well as replacement of venous and urinary catheters and, when clinically indicated, orotracheal intubation tubes. These measures are particularly important because of the risk of secondary device colonization and biofilm formation, which may contribute to persistent bacteremia, metastatic infection, and treatment failure [17,18,19,20].

Despite this, contemporary data on the local susceptibility of *S. aureus* bloodstream isolates in Romania remain limited, and few single-center series describe the resistance profile of bacteraemic isolates under the EUCAST breakpoints now in use. The present two-year period (2024–2025) spans our laboratory’s transition from EUCAST v14.0 to v15.0, and was chosen to provide current, locally relevant susceptibility data to inform empirical therapy and stewardship in our region.

This study aimed primarily to evaluate the antimicrobial susceptibility profile of *Staphylococcus aureus* strains isolated from blood cultures between 2024 and 2025. Secondary aims were to assess the prevalence of MRSA and MDR phenotypes, compare susceptibility patterns between years, describe the clinical characteristics of affected patients, and explore factors associated with unfavorable outcomes.

## 2. Materials and Methods

We performed, on patients with positive blood cultures for *Staphylococcus aureus* (SAB), a single-center longitudinal observational cohort study on patients hospitalized in Sibiu County Clinical Emergency Hospital, Romania. In these analyses, we retrospectively included all consecutive adults (≥18 years) admitted to the Sibiu County Clinical Emergency Hospital, Romania, between 1 January 2024 and 31 December 2025, with positive blood cultures for *Staphylococcus aureus* (SAB). Extracted medical record data included the following variables: year, turnaround time, age, locality, sex, residence setting, number of blood-culture sets, comorbidities and clinical features, phenotypic resistance markers, patient outcome, and antibiotic susceptibility results. Data extraction and consistency checks were performed by all authors. Patients with other positive blood cultures were excluded. From the 314 *S. aureus* blood-culture isolates identified over the study period, we retained a single isolate per patient—the first positive blood culture of the index bacteraemic episode—which yielded the 142 unique patients used for the patient-level analysis. Repeat isolates recovered within the same episode were not counted again [21,22].

Isolates were identified and antimicrobial susceptibility was determined using MALDI-TOF MS for identification and VITEK 2 (Biomerieux Inc., Hazelwood, MO, USA) for susceptibility testing.

For binary analyses and for MDR/XDR derivation, we grouped intermediate and resistant as “non-susceptible”, consistent with standard resistance-phenotype definitions. The S/I/R framework itself follows the standard susceptibility categories used by the European Committee on Antimicrobial Susceptibility Testing, while MDR/XDR definitions follow the standard proposed by Magiorakos and colleagues [23]. Susceptibility categories (S/I/R) were assigned using EUCAST clinical breakpoints in the version current at the time of testing: v14.0 for isolates processed in 2024 and v15.0 for isolates processed in 2025 [21,22].

We performed dataset validation before analysis. We did not impute missing values. Instead, we used available-case denominators for descriptive and pairwise analyses and complete-case analysis for multivariable logistic regression. We defined MRSA phenotypically using cefoxitin positivity and/or the positive MRSA marker for mecA-mediated methicillin resistance. We defined MDR as nonsusceptibility to at least one agent in at least three antimicrobial classes, and XDR as nonsusceptibility to at least one agent in all but two or fewer classes, using the available tested classes in the panel: aminoglycosides, beta-lactams, tetracyclines, folate inhibitors, glycopeptides, fusidanes, fluoroquinolones, oxazolidinones, rifamycins, lincosamides, glycylcyclines, lipopeptides, and macrolides [23]. Unfavorable outcome was defined as in-hospital death; comorbidities were those recorded in the electronic medical record at admission.

For descriptive statistics, we reported counts and percentages for categorical variables and mean ± SD, median [IQR], and range for continuous variables. We assessed distributional shape with Shapiro–Wilk tests. Because turnaround time, age, and number of blood-culture sets harvested per patient were non-normal in both years, year comparisons for continuous variables used the Mann–Whitney U test. Categorical comparisons used chi-square tests or Fisher exact tests when expected cell counts were small. For each antibiotic, we tabulated S/I/R counts and percentages for 2024, 2025, and the overall cohort and compared years using chi-square or Fisher–Freeman–Halton exact tests as appropriate. For pairwise association screens, we used Spearman correlations for ordinal susceptibility outcomes and Mann–Whitney or binary contingency testing for binary nonsusceptibility outcomes. Because the correlation screen involved many simultaneous comparisons, we applied Benjamini–Hochberg false-discovery-rate correction. Finally, we fitted adjusted logistic regression models for mortality, MRSA, and MDR using prespecified covariates: age, sex, hemodialysis status, presence of central venous catheter, presence of malignancies, high blood pressure or other cardiovascular comorbidities, and number of blood-culture sets. The mortality model also included year and turnaround time. We performed all analyses in Python 3.13.5 using pandas 2.2.3, SciPy 1.17.0, statsmodels 0.14.6, and matplotlib 3.10.8.

Associations between susceptibility profiles and a broad set of clinical variables, a fully saturated “all-by-all” inferential framework was not methodologically appropriate for this dataset. With 142 observations, numerous low-frequency covariates, and several antibiotics exhibiting near-zero or zero resistance, an exhaustive correlation and regression matrix would be prone to sparse-data bias, unstable effect estimates, and clinically uninformative signal. Accordingly, the analysis was deliberately focused on: (i) clinically interpretable models for MRSA, MDR, and in-hospital mortality; (ii) pairwise correlations of resistance patterns only among antibiotics with meaningful variability; and (iii) descriptive rather than inferential handling of locality due to fragmentation and limited per-site counts.

**Figure 1:**
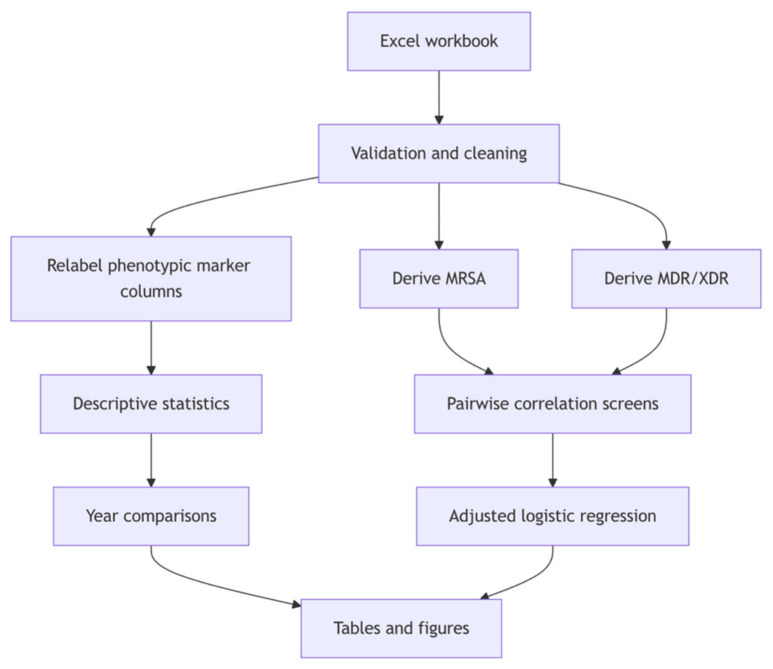
Data validation and statistical analysis workflow.

A structured data-processing workflow was applied prior to statistical analysis (Figure 1).

All participants provided written informed consent prior to inclusion. The study was performed in accordance with the Declaration of Helsinki and received approval from the Institutional Ethics Committee and the Ethics Committee (approval no. 17162; 29 May 2026), which approved the use of anonymized data for publication.

## 3. Results

Of the hospitalized patients in the Sibiu County Clinical Emergency Hospital, Romania, between 1 January 2024 and 31 December 2025, a total of 11,578 blood culture sets were performed, of which 2472 (21.3%) were positive. Among these, 314 isolates (12.7% of positive cultures) were identified as *Staphylococcus aureus*.

The annual distribution showed an increase in microbiological workload and positivity rate over time. In 2024, 5060 blood cultures were processed, yielding 930 positive results (18.4%), including 151 *Staphylococcus aureus* isolates (16.2% of positive cultures). In 2025, the number increased to 6518 blood cultures, with 1542 positive results (23.7%), including 163 *Staphylococcus aureus* isolates (10.6% of positive cultures).

From this pool, 142 positive blood cultures with *Staphylococcus aureus* bacteremia (SAB) from 142 different patients were analyzed.

The cohort of patients was centered on Sibiu and its surrounding areas. Overall, 48.6% of records were from Sibiu city itself, 67.6% were urban, and 55.6% were male. Median age was 70 years, and median turnaround time was 68.7 h.

Part of the continuous descriptive variables analyzed in our study are reported in Table 1.

**Figure 2:**
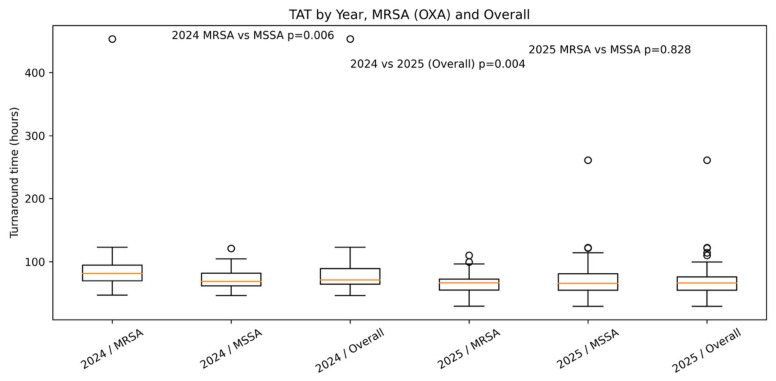
Turnaround time (TAT) expressed in hours, stratified by year, and OXA-defined methicillin resistance status (MRSA vs. MSSA), with overall distributions per year. Boxplots represent median and interquartile range (IQR), with whiskers indicating range and outliers plotted individually. Statistical comparisons were performed using Mann–Whitney U test. In 2024, TAT differed significantly between MRSA and MSSA cases (*p* = 0.006), whereas no significant difference was observed in 2025 (*p* = 0.828). Significant difference in overall TAT distribution was observed between 2024 and 2025 (*p* = 0.004).

Turnaround time was the only continuous variable that changed significantly between years (Figure 2). The direction of change favored 2025, with both lower mean turnaround time and lower median turnaround time. The age of the enrolled patients and the number of blood culture sets harvested per patient were almost identical across years.

In terms of the major comorbidities that have been taken into account for this analysis, they were also broadly similar across the studied years. Overall prevalence was 51.4% for diabetes mellitus, 88.7% for high blood pressure or other cardiovascular comorbidities, 66.0% for acute or chronic kidney disease, 17.6% for coronary angioplasty, 46.5% for the presence of central venous catheters, 9.9% for endocarditis, 16.9% for malignancies, 9.2% for spondylodiscitis, and 20.7% for hemodialysis. The crude outcome distribution in our retrospective analysis is 95 cured (66.9%) and 47 deceased (33.1%). Two categorical comparisons reached significance: high blood pressure or other cardiovascular comorbidities were more common in 2024 than in 2025 (94.3% vs. 83.3%, *p* = 0.039), and malignancy was more common in 2025 (23.6% vs. 10.0%, *p* = 0.03). Central venous catheters were more frequent in 2025 (54.2% vs. 38.6%), but the difference did not reach significance (*p* = 0.062). Our cohort consists of a relatively old population of patients, with a median age of 70 years and an IQR of 61.0–75.8. Part of the profiles of the enrolled patients variables analyzed in our study are reported in Table 2.

Using an operational phenotype-based definition, 56 isolates (39.4%) were classified as MRSA strains. The proportion of MRSA did not differ significantly between 2024 and 2025 (41.4% vs. 37.5%, *p* = 0.632). Applying a category-based operational framework, 70 isolates (49.3%) were classified as MDR, while no isolate met XDR criteria. MDR prevalence was numerically higher in 2025 than in 2024 (54.2% vs. 44.3%), although this difference was not statistically significant (*p* = 0.239).

**Figure 3:**
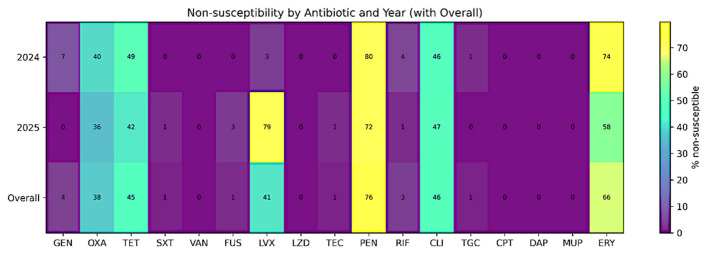
Non-susceptibility rate analysis by year. Gentamicin (GEN), oxacillin (OXA), tetracycline (TET), trimethoprim–sulfamethoxazole (SXT), vancomycin (VAN), fusidic acid (FUS), levofloxacin (LVX), linezolid (LZD), teicoplanin (TEC), penicillin (PEN), rifampicin (RIF), clindamycin (CLI), tigecycline (TGC), ceftaroline (CPT), daptomycin (DAP), mupirocin (MUP), and erythromycin (ERY). The year comparison identifies three antibiotic fields with statistically detectable change: gentamicin, levofloxacin, and erythromycin. The levofloxacin shift is the most clinically conspicuous and should be discussed carefully, because the change was driven largely by a marked increase in intermediate results rather than by outright resistance alone.

The most frequent resistance overall was observed for penicillin (75.9% resistant), erythromycin (66.2% resistant), clindamycin (46.5% resistant), tetracycline (45.1% resistant), and oxacillin (38.0% resistant). In contrast, vancomycin, linezolid, ceftaroline, daptomycin, and mupirocin were fully susceptible in all tested isolates. Year-specific susceptibility was largely stable, with three notable exceptions. Gentamicin susceptibility improved from 92.9% in 2024 to 100.0% in 2025 (*p* = 0.027). Levofloxacin showed a striking distributional shift, from 97.1% susceptible and 2.9% intermediate in 2024 to 21.1% susceptible, 73.2% intermediate, and 5.6% resistant in 2025 (*p* < 0.001). Erythromycin susceptibility increased from 25.7% in 2024 to 41.7% in 2025 (*p* = 0.045). Oxacillin, tetracycline, penicillin, clindamycin, rifampin, teicoplanin, tigecycline, and trimethoprim/sulfamethoxazole did not differ significantly by year. Full results of the antimicrobial susceptibility testing are reported in Table 3 and non-susceptibility rates have also been reported as a heatmap in Figure 3. Methicillin resistance, defined by cefoxitin positivity and/or a positive mecA marker, was marginally more frequent in 2024 than in 2025 (29 vs. 27 isolates); in each year, a single isolate was oxacillin-susceptible but cefoxitin-positive and was therefore classified as MRSA. MLSBi status was present in 48.1% of tested isolates and was also stable between years.

### 3.1. Univariable Associations

In univariable analyses, MDR was positively associated with age (OR per year 1.03, 95% CI 1.01–1.06; *p* = 0.008) and with a history of coronary angioplasty (OR 2.57, 95% CI 1.03–6.41; *p* = 0.044). Turnaround time was not associated with MRSA status (OR per hour 1.01, 95% CI 1.00–1.02; *p* = 0.217). Turnaround time tended to be longer in MRSA-positive than MRSA-negative isolates, median 72.1 vs. 67.4 h. For mortality, age showed a borderline association (OR per year 1.03, 95% CI 1.00–1.05; *p* = 0.054), whereas hemodialysis status was inversely associated with death (OR 0.18, 95% CI 0.05–0.64; *p* = 0.008). Neither MRSA nor MDR status was associated with mortality in unadjusted analyses (MRSA: OR 0.93, 95% CI 0.45–1.91; *p* = 0.845; MDR: OR 0.98, 95% CI 0.49–1.97; *p* = 0.952).

### 3.2. Multivariable Associations

In the adjusted mortality model, acute and chronic kidney disease were independently associated with increased odds of death (adjusted OR 3.83, 95% CI 1.41–10.39; *p* = 0.008). Hemodialysis status remained inversely associated with mortality (adjusted OR 0.11, 95% CI 0.02–0.47; *p* = 0.003), while age demonstrated a non-significant trend (adjusted OR per year 1.03, 95% CI 1.00–1.06; *p* = 0.067). Importantly, MRSA and MDR status were not independently associated with death after adjustment (MRSA: adjusted OR 1.63, 95% CI 0.57–4.68; *p* = 0.364; MDR: adjusted OR 0.56, 95% CI 0.20–1.60; *p* = 0.280). In the adjusted MDR model, endocarditis was inversely associated with MDR (adjusted OR 0.19, 95% CI 0.04–0.86; *p* = 0.031), while coronary angioplasty showed a borderline association (adjusted OR 3.14, 95% CI 0.98–10.09; *p* = 0.054).

### 3.3. Fusidic Acid Resistance—Case-Level Description

Fusidic acid resistance was rare in our cohort, being identified in only 2 out of 142 isolates (1.4%), both recorded in 2025. Both isolates were MRSA with an MDR phenotype, occurred in elderly urban patients with cardiovascular comorbidity, and retained susceptibility to vancomycin, linezolid, daptomycin, and ceftaroline; both patients had spondylodiscitis and a favorable outcome.

## 4. Discussion

In our cohort, acute or chronic renal failure emerged as an independent factor associated with increased mortality. This contrasts with a Dutch multicenter prospective cohort, in which age, comorbidity burden, concomitant endocarditis, and septic shock were independent predictors of mortality, whereas sex, diabetes mellitus, hemodialysis, and immunosuppressive therapy were not significantly associated with 90-day mortality; in that study, the 90-day mortality rate was 33%, and roughly 60% of deaths were directly attributable to staphylococcal infection [24]. This divergence underscores the prognostic heterogeneity of invasive staphylococcal infection and suggests that the impact of renal dysfunction on outcome varies with patient population, infection severity, and comorbidity profile. The same cohort reported a substantial burden of prosthetic implants (9% of patients). Beyond these findings, earlier studies have linked unfavorable outcomes in staphylococcal bacteremia to male sex, an indwelling venous catheter, skin and soft-tissue lesions as the likely portal of entry, advanced age, and metastatic complications such as endocarditis or osteomyelitis [25,26,27]. Biofilm formation in patients with indwelling medical devices plays a pivotal role in the progression and persistence of sepsis. In this setting, combination antimicrobial therapy is often required, alongside timely assessment of the need for device removal or replacement [28,29]. Pending definitive antimicrobial susceptibility results, empirical treatment should include a beta-lactam with antistaphylococcal activity combined with an anti-MRSA agent. Once microbiological data become available, therapy should be reassessed, allowing targeted optimization and de-escalation whenever the susceptibility profile permits [30].

Recognition of infective endocarditis in patients with *Staphylococcus aureus*-positive blood cultures is essential, both for selecting the appropriate therapeutic strategy, in terms of endocardial penetration and duration of antimicrobial therapy, and for identifying cases requiring prompt surgical intervention. Transesophageal echocardiography is considered the preferred diagnostic modality and should be performed as early as possible, ideally immediately after bacteriological confirmation or within the first 3–5 days. When clinical suspicion persists despite an inconclusive initial examination, repeat transesophageal echocardiography after approximately 7 days may be useful [31,32]. Among modern imaging techniques, FDG-PET/CT has emerged as a valuable tool for detecting septic foci and metastatic infectious complications, offering high diagnostic accuracy in selected cases of complicated *S. aureus* bacteremia [33].

A meta-analysis of 341 studies on staphylococcal sepsis (536,791 patients) described a declining mortality trend over the past three decades, with rates of 10% at 7 days, 13% at 2 weeks, 18% at 1 month, 27% at 3 months, and 30% at 1 year [34]. A 2020 study of 31,002 patients within the US Veterans Health Administration reported a 5-year mortality of 61% following an episode of bacteremia [35].

These risk factors for unfavorable outcomes were also observed in the aforementioned study and included advanced age and the presence of comorbidities such as heart failure, alcohol use disorder, malignancy, immunosuppression, and chronic kidney disease requiring hemodialysis. Our study is limited by the lack of systematic post-discharge follow-up; however, it provides relevant data on the in-hospital course of patients with MRSA infection. Notably, MRSA was not significantly associated with mortality in our cohort, a finding that may suggest the effectiveness of first-line therapeutic strategies even against these resistant strains.

The inverse association between hemodialysis and mortality in our adjusted model (aOR 0.11, 95% CI 0.02–0.47) is counter-intuitive and deserves comment, since patients on maintenance dialysis are generally considered high-risk for *S. aureus* bacteremia. Several factors probably contribute. Dialysis patients are in frequent contact with the healthcare system and under close clinical observation, so bacteremia tends to be recognized and treated early. The vascular access is an obvious and, crucially, removable focus, which allows prompt source control—the same logic reflected in cohorts where a non-vascular-access source of infection carried substantially higher mortality than an access-related source [36]. Access-related *S. aureus* bacteremia in this population is also managed along well-worn clinical pathways, with near-reflexive empirical anti-MRSA cover; because vancomycin is largely renally cleared, dialysis patients maintain therapeutic exposure with relatively straightforward dosing [37]. These signals should be read as in-hospital only: we did not capture post-discharge events, and the long-term excess mortality well described in the dialysis population would not be visible here. The small number of hemodialysis patients (n = 29) also widens the confidence interval and warrants caution before firm conclusions are drawn.

From an antimicrobial resistance perspective, over the two-year study period, 38% of the isolates were resistant to oxacillin and 46.5% to clindamycin. Because we did not collect antibiotic-consumption data, we do not attribute this rate to any particular prescribing pattern. Erythromycin resistance, in contrast, declined from 74.3% in 2024 to 58.3% in 2025. Particular attention should be paid to the emergence of fusidic acid resistance among staphylococcal isolates in 2025, which warrants prompt investigation of the underlying resistance mechanisms. Their rarity and clustering in 2025 suggest a sporadic rather than endemic resistance pattern, without evidence of therapeutic compromise in this cohort.

The apparent collapse in levofloxacin susceptibility between 2024 and 2025—from 97.1% to 21.1%—should be read with caution, because its shape does not fit genuine emerging resistance. The change was driven almost entirely by movement into the intermediate category (2.9% to 73.2%), while frank resistance remained negligible (0% to 5.6%). True fluoroquinolone selection pressure would be expected to generate resistant isolates through mutations in the quinolone-resistance-determining regions, not a near-uniform band of intermediate results. Under EUCAST, staphylococci are screened for fluoroquinolone activity using norfloxacin, and wild-type isolates that test susceptible to norfloxacin are reported as “susceptible, increased exposure” (I)—rather than fully susceptible (S)—for levofloxacin and ciprofloxacin [21,22]. A shift of this kind, concentrated in the intermediate band and coinciding with the transition from EUCAST v14.0 (2024) to v15.0 (2025), is therefore best interpreted as a change in interpretive categorization rather than as a real loss of activity. We caution against reading the 2025 levofloxacin susceptibility rate as evidence of rising resistance: the underlying wild-type population appears largely unchanged, and the difference lies in how borderline results were categorized.

Taken together, our findings point to a substantial resistance burden alongside preserved activity of the agents that matter most for salvage. MRSA accounted for 39.4% of isolates and MDR for 49.3%, yet neither was an independent predictor of in-hospital death; acute or chronic kidney disease was. This suggests that outcome in our cohort was driven more by the patient’s comorbidity burden than by the resistance phenotype of the infecting strain, a reading supported by the fully retained in vitro activity of vancomycin, linezolid, daptomycin, and ceftaroline. The practical implication is that empirical anti-MRSA cover remains justified locally given the MRSA prevalence, while the preserved last-line activity offers a dependable route for targeted therapy once susceptibilities return. The two significant year-to-year differences—more cardiovascular comorbidity in 2024 and more malignancy in 2025—are most plausibly explained by variation in cohort and referral composition at a single center over two years, rather than by a genuine secular change in the patient population.

Placed against national data, our resistance profile is broadly consistent with recent Romanian experience. A tertiary-center series from Bucharest covering 2017–2022 reported MRSA in 39.11% of *S. aureus* isolates, almost identical to our 39.4%, together with erythromycin resistance near 50%, inducible clindamycin resistance around 36%, ciprofloxacin resistance close to 10%, and no vancomycin resistance [38]. Macrolide and lincosamide resistance ran somewhat higher in our cohort, but the overall pattern—a high MRSA proportion with fully preserved glycopeptide activity—is shared. This fits EARS-Net surveillance, in which Romania has consistently ranked among the countries with the highest proportion of MRSA among invasive *S. aureus* isolates in the EU/EEA [7].

Because established options remain limited, a broad investigational pipeline is being pursued, although its relevance to our cohort is indirect given the preserved activity of current last-line agents: newer oxazolidinones such as sutezolid, radezolid [39], delpazolid, and Lefamulin; a pleuromutilin antibiotic [40]; and natural-product leads including marine-fungal metabolites from *Stachybotrys* sp. (strain MF347) [41] and antimicrobial peptides such as insect-derived thanatin [42], LL-37 [43], and brilacidin [44].

Complementary approaches act on targets outside the conventional antibiotic repertoire—inhibition of lipoteichoic acid biosynthesis (e.g., HSGN-94 and HSGN-189) [44], and interference with essential enzymes and cell-division machinery such as enoyl–acyl carrier protein reductase (FabI) and the filamenting temperature-sensitive Z protein (FtsZ) [45,46], or combine established antibiotics with efflux-pump, Erm-methyltransferase, or aminoglycoside-modifying-enzyme inhibitors [47], to restore activity against resistant strains [48].

The management of systemic infections caused by *Staphylococcus aureus* can be broadly summarized as (i) pathogen-directed antimicrobial therapy, constrained by multidrug resistance, adequate drug penetration at the site of infection, and treatment-limiting toxicities (notably nephrotoxicity, but also myelotoxicity and cardiovascular adverse effects); (ii) supportive care with organ function monitoring and stabilization; and (iii) surgical management when indicated for source control [49].

This study has several limitations that should be acknowledged. First, it was a retrospective, single-center analysis, performed in one tertiary hospital in Romania, which may limit the generalizability of the findings to other regions or healthcare settings. Local antimicrobial ecology, referral patterns, laboratory workflow, and patient characteristics may have influenced both the resistance profile and clinical outcomes. At the same time, this single-center focus is not only a constraint on external validity but also the source of the study’s practical value, since empirical therapy is best anchored to the resistance ecology of the institution where patients are actually treated, and multicenter aggregates can blur precisely the local signals that guide day-to-day prescribing. Second, although the total microbiological workload was large, the detailed patient-level analysis included 142 cases of *Staphylococcus aureus* bacteremia, which limits the statistical power for subgroup analyses, particularly for low-frequency events such as endocarditis, spondylodiscitis, fusidic acid resistance, XDR phenotypes, and resistance to last-line agents. Third, the outcome variable reflected crude in-hospital evolution, categorized as cured or deceased. Consequently, mortality analysis may have been affected by residual confounding. We also did not record the empirical or targeted antimicrobial therapy actually administered, collected no antibiotic-consumption data, and had no systematic post-discharge follow-up, so late events and attributable mortality could not be assessed. Despite these limitations, the study provides valuable real-world data on the antimicrobial susceptibility profile of *S. aureus* bloodstream isolates and highlights the importance of continued local surveillance to guide empirical and targeted therapy.

## 5. Conclusions

*Staphylococcus aureus* bacteremia was predominantly observed in patients with underlying comorbidities, including diabetes mellitus, cardiovascular disease, renal impairment, and malignancy, as well as in those with indwelling medical devices, particularly central venous catheters, which were present in 46.5% of cases. The overall mortality rate was 33.1%. MRSA accounted for 39.4% of isolates and MDR for 49.3%, while vancomycin, linezolid, ceftaroline, daptomycin, and mupirocin retained full activity; acute or chronic kidney disease, rather than MRSA or MDR status, was independently associated with mortality. Because treatment efficacy was not evaluated, we draw no conclusion about optimal regimens; the findings do, however, underline the value of continued local surveillance, rapid microbiological diagnosis, and stewardship to guide empirical and targeted therapy.

## Figures and Tables

**Table 1:** Continuous descriptive summary of variables.

Variable	Overall	2024	2025	*p*=
Turnaround time, h	76.01 ± 40.64; 68.73 [60.38–86.02]; range 29.54–453.21	81.80 ± 48.55; 71.42 [64.62–89.31]	70.38 ± 30.40; 66.31 [54.97–76.16]	0.004
Age, y	66.56 ± 15.49; 70.00 [61.00–75.75]; range 15.00–91.00	67.00 ± 15.06; 69.50 [61.00–75.75]	66.12 ± 15.99; 70.00 [61.75–75.25]	0.953
Blood culture sets	2.08 ± 0.97; 2.00 [2.00–2.00]; range 1.00–9.00	1.97 ± 0.76; 2.00 [2.00–2.00]	2.19 ± 1.13; 2.00 [2.00–2.00]	0.174

Turnaround time is defined as the time since the blood cultures were sent to the laboratory and the final time of validation of the results and antimicrobial susceptibility test results.

**Table 2:** Year-stratified profile of enrolled patients.

Variable	2024	2025	*p*=
Male sex	36/70 (51.4%)	43/72 (59.7%)	0.32
Urban residence	48/70 (68.6%)	48/72 (66.7%)	0.808
Diabetes	34/70 (48.6%)	39/72 (54.2%)	0.505
High blood pressure or other cardiovascular comorbidities	66/70 (94.3%)	60/72 (83.3%)	0.039
Acute or chronic kidney disease	45/70 (64.2%)	48/72 (66.7%)	0.856
Cured at discharge	45/70 (64.3%)	50/72 (69.4%)	0.514
Coronary angioplasty	11/70 (15.7%)	14/72 (19.4%)	0.56
Central venous catheters	27/70 (38.6%)	39/72 (54.2%)	0.062
Endocarditis	7/70 (10.0%)	7/72 (9.7%)	0.956
Malignancies	7/70 (10.0%)	17/72 (23.6%)	0.03
Spondylodiscitis	6/70 (8.6%)	7/72 (9.7%)	0.812
Hemodialysis	13/70 (18.57%)	16/72 (22.2%)	0.651
MRSA strain	29/70 (41.4%)	27/72 (37.5%)	0.632
MDR isolated strains	31/70 (44.3%)	39/72 (54.2%)	0.239
XDR isolated strains	0/70 (0.0%)	0/72 (0.0%)	1

**Table 3:** Susceptibility tables by year.

Antibiotic	N Tested	Total S/I/R	2024 S/I/R	2025 S/I/R	*p*=
GEN	142	137/142 (96.5%); 0/142 (0.0%); 5/142 (3.5%)	65/70 (92.9%); 0/70 (0.0%); 5/70 (7.1%)	72/72 (100.0%); 0/72 (0.0%); 0/72 (0.0%)	0.027
OXA	142	88/142 (62.0%); 0/142 (0.0%); 54/142 (38.0%)	42/70 (60.0%); 0/70 (0.0%); 28/70 (40.0%)	46/72 (63.9%); 0/72 (0.0%); 26/72 (36.1%)	0.633
TET	142	78/142 (54.9%); 0/142 (0.0%); 64/142 (45.1%)	36/70 (51.4%); 0/70 (0.0%); 34/70 (48.6%)	42/72 (58.3%); 0/72 (0.0%); 30/72 (41.7%)	0.408
SXT	142	141/142 (99.3%); 0/142 (0.0%); 1/142 (0.7%)	70/70 (100.0%); 0/70 (0.0%); 0/70 (0.0%)	71/72 (98.6%); 0/72 (0.0%); 1/72 (1.4%)	1
VAN	142	142/142 (100.0%); 0/142 (0.0%); 0/142 (0.0%)	70/70 (100.0%); 0/70 (0.0%); 0/70 (0.0%)	72/72 (100.0%); 0/72 (0.0%); 0/72 (0.0%)	NA
FUS	142	140/142 (98.6%); 0/142 (0.0%); 2/142 (1.4%)	70/70 (100.0%); 0/70 (0.0%); 0/70 (0.0%)	70/72 (97.2%); 0/72 (0.0%); 2/72 (2.8%)	0.497
LVX	141	83/141 (58.9%); 54/141 (38.3%); 4/141 (2.8%)	68/70 (97.1%); 2/70 (2.9%); 0/70 (0.0%)	15/71 (21.1%); 52/71 (73.2%); 4/71 (5.6%)	<0.001
LZD	142	142/142 (100.0%); 0/142 (0.0%); 0/142 (0.0%)	70/70 (100.0%); 0/70 (0.0%); 0/70 (0.0%)	72/72 (100.0%); 0/72 (0.0%); 0/72 (0.0%)	NA
TEC	142	141/142 (99.3%); 0/142 (0.0%); 1/142 (0.7%)	70/70 (100.0%); 0/70 (0.0%); 0/70 (0.0%)	71/72 (98.6%); 0/72 (0.0%); 1/72 (1.4%)	1
PEN	142	34/142 (24.1%); 0/142 (0.0%); 107/142 (75.9%)	14/70 (20.3%); 0/70 (0.0%); 55/70 (79.7%)	20/72 (27.8%); 0/72 (0.0%); 52/72 (72.2%)	0.299
RIF	142	138/142 (97.2%); 0/142 (0.0%); 4/142 (2.8%)	67/70 (95.7%); 0/70 (0.0%); 3/70 (4.3%)	71/72 (98.6%); 0/72 (0.0%); 1/72 (1.4%)	0.363
CLI	142	76/142 (53.5%); 0/142 (0.0%); 66/142 (46.5%)	38/70 (54.3%); 0/70 (0.0%); 32/70 (45.7%)	38/72 (52.8%); 0/72 (0.0%); 34/72 (47.2%)	0.857
TGC	142	141/142 (99.3%); 0/142 (0.0%); 1/142 (0.7%)	69/70 (98.6%); 0/70 (0.0%); 1/70 (1.4%)	72/72 (100.0%); 0/72 (0.0%); 0/72 (0.0%)	0.493
CPT	142	142/142 (100.0%); 0/142 (0.0%); 0/142 (0.0%)	70/70 (100.0%); 0/70 (0.0%); 0/70 (0.0%)	72/72 (100.0%); 0/72 (0.0%); 0/72 (0.0%)	NA
DAP	142	142/142 (100.0%); 0/142 (0.0%); 0/142 (0.0%)	70/70 (100.0%); 0/70 (0.0%); 0/70 (0.0%)	72/72 (100.0%); 0/72 (0.0%); 0/72 (0.0%)	NA
MUP	142	142/142 (100.0%); 0/142 (0.0%); 0/142 (0.0%)	70/70 (100.0%); 0/70 (0.0%); 0/70 (0.0%)	72/72 (100.0%); 0/72 (0.0%); 0/72 (0.0%)	NA
ERY	142	48/142 (33.8%); 0/142 (0.0%); 94/142 (66.2%)	18/70 (25.7%); 0/70 (0.0%); 52/70 (74.3%)	30/72 (41.7%); 0/72 (0.0%); 42/72 (58.3%)	0.045

Antimicrobial susceptibility testing was performed for the following agents: gentamicin (GEN), oxacillin (OXA), tetracycline (TET), trimethoprim–sulfamethoxazole (SXT), vancomycin (VAN), fusidic acid (FUS), levofloxacin (LVX), linezolid (LZD), teicoplanin (TEC), penicillin (PEN), rifampicin (RIF), clindamycin (CLI), tigecycline (TGC), ceftaroline (CPT), daptomycin (DAP), mupirocin (MUP), and erythromycin (ERY). The year comparison identifies three antibiotic fields with statistically detectable change: gentamicin, levofloxacin, and erythromycin.

## Data Availability

The original contributions presented in this study are included in the article. Further inquiries can be directed to the corresponding authors.

## Abbreviations

AST	Antimicrobial susceptibility testing
CA-MRSA	Community-associated MRSA
CI	Confidence interval
CLI	Clindamycin
CPT	Ceftaroline
DAP	Daptomycin
ECDC	European Centre for Disease Prevention and Control
ERY	Erythromycin
EU/EEA	European Union/European Economic Area
EUCAST	European Committee on Antimicrobial Susceptibility Testing
FabI	Enoyl-acyl carrier protein reductase
FUS	Fusidic acid
FtsZ	Filamenting temperature-sensitive Z protein
GEN	Gentamicin
HA-MRSA	Healthcare-associated MRSA
IQR	Interquartile range
LVX	Levofloxacin
LZD	Linezolid
MDR	Multidrug-resistant
MLSBi	Macrolide-lincosamide-streptogramin B inducible
MODSA	Modified *Staphylococcus aureus*
MRSA	Methicillin-resistant *Staphylococcus aureus*
MSSA	Methicillin-susceptible *Staphylococcus aureus*
MUP	Mupirocin
OXA	Oxacillin
OR	Odds ratio
*p*	*p*-value
PEN	Penicillin
RIF	Rifampicin
*S. aureus*	*Staphylococcus aureus*
SAB	*Staphylococcus aureus* bacteremia
S/I/R	Susceptible/intermediate/resistant
SD	Standard deviation
SXT	Trimethoprim-sulfamethoxazole
TAT	Turnaround time
TEC	Teicoplanin
TET	Tetracycline
TGC	Tigecycline
VAN	Vancomycin
WHO	World Health Organization
XDR	Extensively drug-resistant

## References

[1] Kimmig A., Hagel S., Weis S., Bahrs C., Löffler B., Pletz M.W. (2021). Management of *Staphylococcus aureus* Bloodstream Infections. Front. Med..

[2] Laupland K.B., Lyytikäinen O., Sgaard M., Kennedy K.J., Knudsen J.D., Ostergaard C., Galbraith J.C., Valiquette L., Jacobsson G., Collignon P. (2013). The Changing Epidemiology of *Staphylococcus aureus* Bloodstream Infection: A Multinational Population-Based Surveillance Study. Clin. Microbiol. Infect..

[3] Nambiar K., Seifert H., Rieg S., Kern W.V., Scarborough M., Gordon N.C., Kim H.B., Song K.-H., Tilley R., Gott H. (2018). Survival Following *Staphylococcus aureus* Bloodstream Infection: A Prospective Multinational Cohort Study Assessing the Impact of Place of Care. J. Infect..

[4] Thwaites G.E., Scarborough M., Szubert A., Nsutebu E., Tilley R., Greig J., Wyllie S.A., Wilson P., Auckland C., Cairns J. (2018). Adjunctive Rifampicin for *Staphylococcus aureus* Bacteraemia (ARREST): A Multicentre, Randomised, Double-Blind, Placebo-Controlled Trial. Lancet.

[5] World Health Organization (2024). WHO Bacterial Priority Pathogens List, 2024: Bacterial Pathogens of Public Health Importance to Guide Research, Development and Strategies to Prevent and Control Antimicrobial Resistance.

[6] World Health Organization (2022). 2022 Global Antimicrobial Resistance and Use Surveillance System (GLASS) Report 2022.

[7] ECDC Antimicrobial Resistance in the EU/EEA (EARS-Net) Annual Epidemiological Report for 2024. https://www.ecdc.europa.eu/sites/default/files/documents/antimicrobial-resistance-eu-annual-epidemiological-report-2024.pdf#:~:text=In%202024%2C%20the%20estimated%20total%20EU%20incidence,detected%20between%202019%20(baseline%20year)%20and%202024.

[8] Birlutiu V., Birlutiu R.-M. (2025). An Overview of the Epidemiology of Multidrug Resistance and Bacterial Resistance Mechanisms: What Solutions Are Available? A Comprehensive Review. Microorganisms.

[9] Garzoni C., Kelley W.L. (2009). *Staphylococcus aureus*: New Evidence for Intracellular Persistence. Trends Microbiol..

[10] Strobel M., Pförtner H., Tuchscherr L., Völker U., Schmidt F., Kramko N., Schnittler H.-J., Fraunholz M.J., Löffler B., Peters G. (2016). Post-Invasion Events after Infection with *Staphylococcus aureus* Are Strongly Dependent on Both the Host Cell Type and the Infecting *S. aureus* Strain. Clin. Microbiol. Infect..

[11] Tsuji B.T., Rybak M.J., Cheung C.M., Amjad M., Kaatz G.W. (2007). Community- and Health Care-Associated Methicillin-Resistant *Staphylococcus aureus*: A Comparison of Molecular Epidemiology and Antimicrobial Activities of Various Agents. Diagn. Microbiol. Infect. Dis..

[12] David M.Z., Daum R.S. (2010). Community-Associated Methicillin-Resistant *Staphylococcus aureus*: Epidemiology and Clinical Consequences of an Emerging Epidemic. Clin. Microbiol. Rev..

[13] Peacock S.J., Paterson G.K. (2015). Mechanisms of Methicillin Resistance in *Staphylococcus aureus*. Annu. Rev. Biochem..

[14] González-López A., Selmer M. (2025). Mechanisms of Fusidic Acid Resistance. Biochem. Soc. Trans..

[15] Asgeirsson H., Thalme A., Weiland O. (2018). *Staphylococcus aureus* Bacteraemia and Endocarditis—Epidemiology and Outcome: A Review. Infect. Dis..

[16] Lopez-Cortes L.E., del Toro M.D., Galvez-Acebal J., Bereciartua-Bastarrica E., Farinas M.C., Sanz-Franco M., Natera C., Corzo J.E., Lomas J.M., Pasquau J. (2013). Impact of an Evidence-Based Bundle Intervention in the Quality-of-Care Management and Outcome of *Staphylococcus aureus* Bacteremia. Clin. Infect. Dis..

[17] Peacock S.J., Eddleston M., Emptage A., King A., Crook D.W.M. (1998). Positive Intravenous Line Tip Cultures as Predictors of Bacteraemia. J. Hosp. Infect..

[18] Mermel L.A., Farr B.M., Sherertz R.J., Raad I.I., O’Grady N., Harris J.S., Craven D.E. (2001). Guidelines for the Management of Intravascular Catheter-Related Infections. Clin. Infect. Dis..

[19] Ekkelenkamp M.B., van der Bruggen T., van de Vijver D.A.M.C., Wolfs T.F.W., Bonten M.J.M. (2008). Bacteremic Complications of Intravascular Catheters Colonized with *Staphylococcus aureus*. Clin. Infect. Dis..

[20] Böll B., Schalk E., Buchheidt D., Hasenkamp J., Kiehl M., Kiderlen T.R., Kochanek M., Koldehoff M., Kostrewa P., Claßen A.Y. (2021). Central Venous Catheter–Related Infections in Hematology and Oncology: 2020 Updated Guidelines on Diagnosis, Management, and Prevention by the Infectious Diseases Working Party (AGIHO) of the German Society of Hematology and Medical Oncology (DGHO). Ann. Hematol..

[21] European Committee on Antimicrobial Susceptibility Testing (2025). Clinical Breakpoints and Interpretation of Clinical MICs, Version 15.

[22] European Committee on Antimicrobial Susceptibility Testing (2024). Clinical Breakpoints and Interpretation of Clinical MICs, Version 14.

[23] Magiorakos A.-P., Srinivasan A., Carey R.B., Carmeli Y., Falagas M.E., Giske C.G., Harbarth S., Hindler J.F., Kahlmeter G., Olsson-Liljequist B. (2012). Multidrug-Resistant, Extensively Drug-Resistant and Pandrug-Resistant Bacteria: An International Expert Proposal for Interim Standard Definitions for Acquired Resistance. Clin. Microbiol. Infect..

[24] van der Vaart T.W., Prins J.M., Soetekouw R., van Twillert G., Veenstra J., Herpers B.L., Rozemeijer W., Jansen R.R., Bonten M.J.M., van der Meer J.T.M. (2022). All-Cause and Infection-Related Mortality in *Staphylococcus aureus* Bacteremia, a Multicenter Prospective Cohort Study. Open Forum Infect. Dis..

[25] Le Moing V., Alla F., Doco-Lecompte T., Delahaye F., Piroth L., Chirouze C., Tattevin P., Lavigne J.-P., Erpelding M.-L., Hoen B. (2015). *Staphylococcus aureus* Bloodstream Infection and Endocarditis—A Prospective Cohort Study. PLoS ONE.

[26] Ariaans M.B.P.A., Roovers E.A., Claassen M.A.A., Hassing R.-J., Swanink C.M.A., Gisolf E.H. (2018). Increased Overall Survival after Introduction of Structured Bedside Consultation in *Staphylococcus aureus* Bacteraemia. Eur. J. Clin. Microbiol. Infect. Dis..

[27] Kuehl R., Morata L., Boeing C., Subirana I., Seifert H., Rieg S., Kern W.V., Kim H.B., Kim E.S., Liao C.-H. (2020). Defining Persistent *Staphylococcus aureus* Bacteraemia: Secondary Analysis of a Prospective Cohort Study. Lancet Infect. Dis..

[28] Birlutiu R.-M., Birlutiu V. (2025). Oritavancin a Therapeutic Option for Periprosthetic Joint Infections in Selected Cases: A Comprehensive Review. Pharmaceuticals.

[29] Dragosloveanu S., Birlutiu R.-M., Neamtu B., Birlutiu V. (2025). Microbiological Profiles, Antibiotic Susceptibility Patterns and the Role of Multidrug-Resistant Organisms in Patients Diagnosed with Periprosthetic Joint Infection over 8 Years: Results from a Single-Center Observational Cohort Study from Romania. Microorganisms.

[30] Lam J.C., Stokes W. (2023). The Golden Grapes of Wrath—*Staphylococcus aureus* Bacteremia: A Clinical Review. Am. J. Med..

[31] Habib G., Lancellotti P., Antunes M.J., Bongiorni M.G., Casalta J.-P., Del Zotti F., Dulgheru R., El Khoury G., Erba P.A., Iung B. (2015). 2015 ESC Guidelines for the Management of Infective Endocarditis. Eur. Heart J..

[32] Palraj B.R., Baddour L.M., Hess E.P., Steckelberg J.M., Wilson W.R., Lahr B.D., Sohail M.R. (2015). Predicting Risk of Endocarditis Using a Clinical Tool (PREDICT): Scoring System to Guide Use of Echocardiography in the Management of *Staphylococcus aureus* Bacteremia. Clin. Infect. Dis..

[33] Vos F.J., Kullberg B.J., Sturm P.D., Krabbe P.F.M., van Dijk A.P.J., Wanten G.J.A., Oyen W.J.G., Bleeker-Rovers C.P. (2012). Metastatic Infectious Disease and Clinical Outcome in *Staphylococcus aureus* and *Streptococcus* Species Bacteremia. Medicine.

[34] Bai A.D., Lo C.K.L., Komorowski A.S., Suresh M., Guo K., Garg A., Tandon P., Senecal J., Del Corpo O., Stefanova I. (2022). *Staphylococcus aureus* Bacteraemia Mortality: A Systematic Review and Meta-Analysis. Clin. Microbiol. Infect..

[35] Goto M., Jones M.P., Schweizer M.L., Livorsi D.J., Perencevich E.N., Richardson K., Beck B.F., Alexander B., Ohl M.E. (2020). Association of Infectious Diseases Consultation with Long-Term Postdischarge Outcomes Among Patients with *Staphylococcus aureus* Bacteremia. JAMA Netw. Open.

[36] Sinclair M.R., Souli M., Ruffin F., Park L.P., Dagher M., Eichenberger E.M., Maskarinec S.A., Thaden J.T., Mohnasky M., Wyatt C.M. (2022). *Staphylococcus aureus* Bacteremia Among Patients Receiving Maintenance Hemodialysis: Trends in Clinical Characteristics and Outcomes. Am. J. Kidney Dis..

[37] Inrig J.K., Reed S.D., Szczech L.A., Engemann J.J., Friedman J.Y., Corey G.R., Schulman K.A., Reller L.B., Fowler V.G. (2006). Relationship between Clinical Outcomes and Vascular Access Type among Hemodialysis Patients with *Staphylococcus aureus* Bacteremia. Clin. J. Am. Soc. Nephrol..

[38] Tălăpan D., Sandu A.-M., Rafila A. (2023). Antimicrobial Resistance of *Staphylococcus aureus* Isolated between 2017 and 2022 from Infections at a Tertiary Care Hospital in Romania. Antibiotics.

[39] Foti C., Piperno A., Scala A., Giuffrè O. (2021). Oxazolidinone Antibiotics: Chemical, Biological and Analytical Aspects. Molecules.

[40] Zhanel G.G., Deng C., Zelenitsky S., Lawrence C.K., Adam H.J., Golden A., Berry L., Schweizer F., Zhanel M.A., Irfan N. (2021). Lefamulin: A Novel Oral and Intravenous Pleuromutilin for the Treatment of Community-Acquired Bacterial Pneumonia. Drugs.

[41] Liu M., El-Hossary E.M., Oelschlaeger T.A., Donia M.S., Quinn R.J., Abdelmohsen U.R. (2019). Potential of Marine Natural Products against Drug-Resistant Bacterial Infections. Lancet Infect. Dis..

[42] Dash R., Bhattacharjya S. (2021). Thanatin: An Emerging Host Defense Antimicrobial Peptide with Multiple Modes of Action. Int. J. Mol. Sci..

[43] Piller P., Wolinski H., Cordfunke R.A., Drijfhout J.W., Keller S., Lohner K., Malanovic N. (2022). Membrane Activity of LL-37 Derived Antimicrobial Peptides against *Enterococcus hirae*: Superiority of SAAP-148 over OP-145. Biomolecules.

[44] Gajdács M. (2019). The Continuing Threat of Methicillin-Resistant *Staphylococcus aureus*. Antibiotics.

[45] Bibens L., Becker J.-P., Dassonville-Klimpt A., Sonnet P. (2023). A Review of Fatty Acid Biosynthesis Enzyme Inhibitors as Promising Antimicrobial Drugs. Pharmaceuticals.

[46] Tripathy S., Sahu S.K. (2019). FtsZ Inhibitors as a New Genera of Antibacterial Agents. Bioorg. Chem..

[47] Magaña A.J., Sklenicka J., Pinilla C., Giulianotti M., Chapagain P., Santos R., Ramirez M.S., Tolmasky M.E. (2023). Restoring Susceptibility to Aminoglycosides: Identifying Small Molecule Inhibitors of Enzymatic Inactivation. RSC Med. Chem..

[48] Brdová D., Ruml T., Viktorová J. (2024). Mechanism of Staphylococcal Resistance to Clinically Relevant Antibiotics. Drug Resist. Updates.

[49] Kwiecinski J.M., Horswill A.R. (2020). *Staphylococcus aureus* Bloodstream Infections: Pathogenesis and Regulatory Mechanisms. Curr. Opin. Microbiol..

